# Supplementary material for: Antiferromagnetic Skyrmion: Stability, Creation and Manipulation
Source: Sci Rep. 2016 Apr 21;6:24795. doi: 10.1038/srep24795 (PMC4838875; doi:10.1038/srep24795)
Supplement: Supplementary Information [file srep24795-s1.pdf]

Antiferromagnetic skyrmion: stability, creation and manipulation

Xichao Zhang[1, 2], Yan Zhou[1, 2, *], Motohiko Ezawa[3, †]

1. Department of Physics, University of Hong Kong, Hong Kong, China

2. School of Electronics Science and Engineering, Nanjing University, Nanjing 210093, China

3. Department of Applied Physics, University of Tokyo, Hongo 7-3-1, Tokyo 113-8656, Japan

*E-mail: yanzhou@hku.hk

†E-mail: ezawa@ap.t.u-tokyo.ac.jp

SUPPLEMENTARY INFORMATION

Supplementary Figures

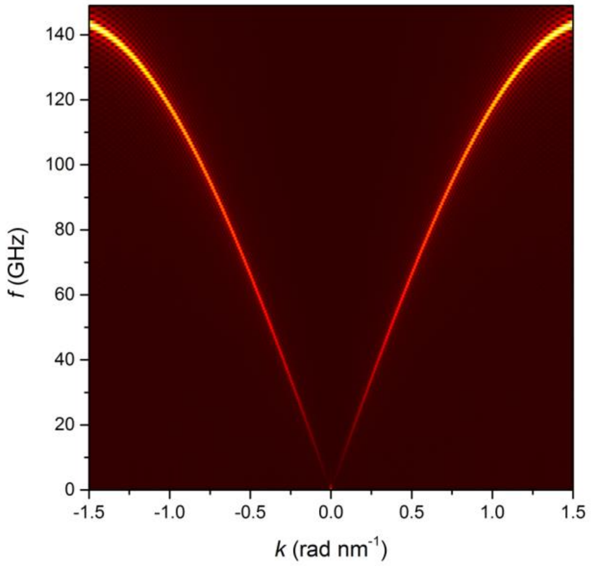

Supplementary Figure 1. Linear dispersion relation of the AFM spin wave based on the lattice Hamiltonian (see equation (1) in the main text) together with D = 0 and K = 0. We have numerically calculated the dispersion relation of spin wave propagation in the 1-dimensional antiferromagnetic chain by using OOMMF. The chain is 2000-nm-long, which contains 2000 antiferromagnetically exchange-coupled spins along y-direction. A local external magnetic field pulse with the profile of the cardinal sine function is applied at the center of the chain to excite spin waves, which propagate along the length direction (x-direction) of chain. The dispersion curves are obtained by performing the Fourier transformation of the x-component magnetization in space and time with contributions from all the spins.

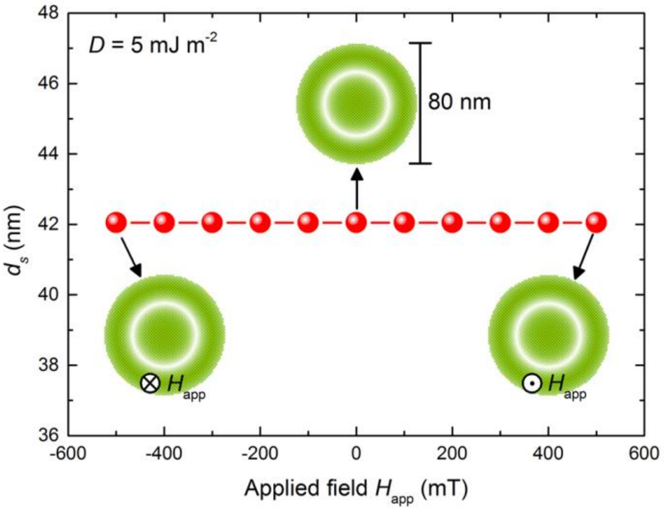

Supplementary Figure 2. AFM skyrmion size (d_s) as a function of the applied field H_{app} perpendicular to the nanodisk. The diameter of the nanodisk is 80 nm and the thickness is 0.4 nm. Here, we use $D = 5\times10^{-21}$ J.

Supplementary Movie Captions

Supplementary Movie 1. Creation of an AFM skyrmion in a nanodisk via spin-polarized current injected perpendicularly to the nanodisk. A 2-ns-long spin-polarized ($P = 0.4$, along -z) current pulse with current density $j = 3\times10^{13}$ A m^{-2} is perpendicularly injected into the 80-nm-diameter and 0.4-nm-thick nanodisk followed by a 1-ns-long relaxation. Here, we use $D = 6\times10^{-21}$ J. The nanodisk is the AFM ground state at initial. The diameter of the spin-polarized current injection region equals 40 nm.

Supplementary Movie 2. Creation of an AFM skyrmion in a nanodisk via spin-polarized current injected perpendicularly to the nanodisk. A 0.5-ns-long spin-polarized ($P = 0.4$, along -z) current pulse with current density $j = 10\times10^{13}$ A m^{-2} is perpendicularly injected into the 80-nm-diameter and 0.4-nm-thick nanodisk followed by a 2.5-ns-long relaxation. Here, we use $D = 5.5\times10^{-21}$ J. The nanodisk is the AFM ground state at initial. The diameter of the spin-polarized current injection region equals 30 nm. See also Supplementary Movie 8. The movies demonstrate the AFM skyrmion will relax to stable size once it is created, no matter whether the injection region is larger or smaller than the stable size, denoting the stability of the AFM skyrmion.

Supplementary Movie 3. Creation of two AFM skyrmions in a nanotrack via out-of-plane current pulses. Two 10-ps-long spin-polarized ($P = 0.4$) current pulses with current density $j = 5\times10^{13}$ A m^{-2} are perpendicularly injected into two 40-nm-diameter circle regions followed

by a 40-ps-long relaxation, of which the regions are denoted by the blue circles. The left side current pulse is polarized along $-z$, while the right side pulse is polarized along $+z$. The nanotrack (width = 100 nm, thickness = 0.4 nm) is of the AFM ground state at initial. Here, we use $D = 4\times10^{-21}$ J.

Supplementary Movie 4. Creation of an AFM skyrmion in a nanotrack via AFM domain wall pair driven by vertical current. A vertical current with density $j = 4.5\times10^{11}$ A m^{-2} in wide part is applied perpendicular to the nanotrack from bottom. The current density inside the wide part of the nanotrack is proportional to the current density inside the narrow part of the nanotrack with a ratio of narrow proportion (100 nm × 20 nm) to wide proportion (100 nm × 100 nm). Here, we use $D = 4\times10^{-21}$ J. The AFM skyrmion is created from an antiferromagnetic domain wall pair driven by the current moving from the narrow part to the wide part of the nanotrack. The antiferromagnetic domain wall pair can be created by locally applied vertical spin current/magnetic field.

Supplementary Movie 5. High-speed motion of an AFM skyrmion chain with encoded information (11101101) in a 4000-nm-long nanotrack driven by vertical current ($j = 100\times10^{10}$ A m^{-2}, $P = 0.4$, polarized along $-y$). Here, we use $D = 3.5\times10^{-21}$ J.

Supplementary Movie 6. Steady marching of AFM skyrmions in a 400-nm long and wide film driven by vertical current ($j = 10\times10^{10}$ A m^{-2}, $P = 0.4$, polarized along $-y$). Here, we use $D = 4\times10^{-21}$ J.

Supplementary Movie 7. Motion and destruction of FM skyrmions in a 400-nm long and wide film driven by vertical current ($j = 10\times10^{10}$ A m^{-2}, $P = 0.4$, polarized along y). Here, we use $D = 3.5\times10^{-21}$ J.
