# Supplementary figures and images for: Antiferromagnetic Skyrmion: Stability, Creation and Manipulation
Source: Sci Rep. 2016 Apr 21;6:24795. doi: 10.1038/srep24795 (PMC4838875; doi:10.1038/srep24795)

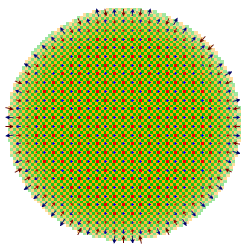

Supplement: Supplementary Movie 1 [file srep24795-s2.gif]

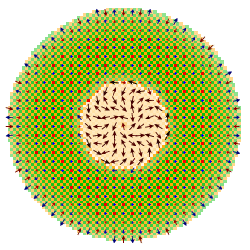

Supplement: Supplementary Movie 2 [file srep24795-s3.gif]

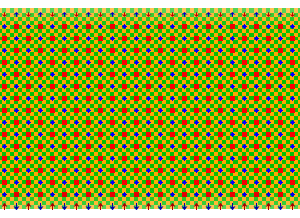

Supplement: Supplementary Movie 3 [file srep24795-s4.gif]

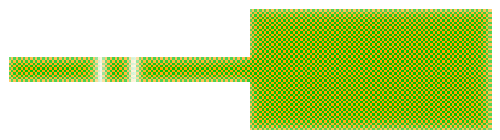

Supplement: Supplementary Movie 4 [file srep24795-s5.gif]

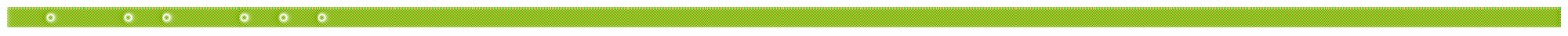

Supplement: Supplementary Movie 5 [file srep24795-s6.gif]

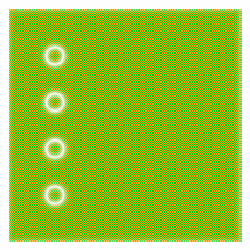

Supplement: Supplementary Movie 6 [file srep24795-s7.gif]

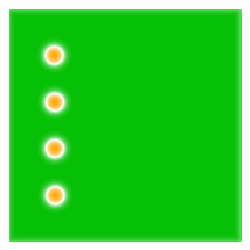

Supplement: Supplementary Movie 7 [file srep24795-s8.gif]
